# Elevated Red Cell Distribution Width Is Independently Associated With a Higher Frailty Risk Among 2,932 Community-Dwelling Older Adults

**DOI:** 10.3389/fmed.2020.00470

**Published:** 2020-08-25

**Authors:** Chia-Ming Li, Chia-Ter Chao, Shih-I Chen, Der-Sheng Han, Kuo-Chin Huang

**Affiliations:** ^1^Department of Family Medicine, National Taiwan University Hospital BeiHu Branch, Taipei, Taiwan; ^2^Geriatric and Community Medicine Research Center, National Taiwan University Hospital BeiHu Branch, Taipei, Taiwan; ^3^Nephrology Division, Department of Internal Medicine, National Taiwan University Hospital BeiHu Branch, Taipei, Taiwan; ^4^Graduate Institute of Toxicology, National Taiwan University, Taipei, Taiwan; ^5^Department of Rehabilitation and Physical Medicine, National Taiwan University Hospital BeiHu Branch, Taipei, Taiwan; ^6^Department of Family Medicine, National Taiwan University Hospital, Taipei, Taiwan

**Keywords:** geriatric, frailty, frail phenotype, red cell distribution width, study of osteoporotic fractures

## Abstract

**Background:** Older adults are at an increased risk of frailty, but laboratory surrogates for identifying frailty in this population remain controversial and clinicians frequently encounter difficulty during frailty screening. We examined whether having a high red cell distribution width (RDW) was associated with an increased probability of frailty in older adults.

**Methods:** We prospectively included community-dwelling older adults between 2013 and 2016 from a single institute, with their clinical features/laboratory parameters documented. We used the Study of Osteoporotic Fractures index (malnutrition, poor physical performance, and fatigue) to delineate frailty, and harnessed multiple logistic regression to investigate whether having a high RDW (≥ 15.7%) was associated with an increased risk of having frailty among these participants.

**Results:** A total of 2,932 older adults (mean 73.5 ± 6.7 years; 44.6% male) were included, among whom 113 (3.9%) and 76 (2.6%) had a high RDW and presented frailty, respectively. Older adults with a high RDW were more likely to be frail (*p* = 0.002) and had more positive SOF items than those with normal RDW levels (*p* = 0.013). Those with a high RDW exhibited a significantly higher risk of having frailty (odds ratio [OR] 2.689, 95% confidence interval [CI] 1.184–6.109) compared to those without. Sensitivity analyses using RDW as a continuous variable similarly showed that RDW levels were positively associated with frailty risk (OR 1.223 per 1% RDW higher).

**Conclusions:** In older adults, higher RDW can be regarded as a frailty indicator, and the readiness in RDW assessment supports its screening utility.

## Background

Frailty is a degenerative phenotype occurring in conjunction with chronological aging, in the form of cumulative subclinical health deficits as well as a compromised physical capacity (1). Older adults are the most severely affected population; moreover, the construct of frailty has now been deemed applicable to those with premature aging phenotypes such as patients with diabetes mellitus (DM), chronic inflammation, and chronic kidney disease (CKD) (2). The presence of frailty is predictive of a substantially elevated risk of adverse outcomes, including mortality, hospitalization, institutionalization, incident disability, and even cardiovascular events (3–5). The prognostic importance of frailty in geriatric population cannot be over-stated, and the quest for risk factors of frailty assumes importance both from the epidemiologic and the clinical perspectives.

Different types of risk factors of frailty have been uncovered to date, ranging from clinical features to laboratory parameters. Red cell distribution width (RDW), a descriptor of red blood cell (RBC) volumetric alterations, estimates the degree of RBC anisocytosis, or the variation in RBC volumes. RBC volume usually lies between 80 and 100 fL, and pathologies involving erythropoiesis may result in progressive fluctuations of RBC volumes as well as sizes, thereby increasing RDW. RDW is normally obtained by dividing the mean corpuscular volume (MCV) standard deviation by the MCV level (6), and is regularly reported in the complete blood count panel. Traditionally much emphasis has been placed on its role in facilitating the differential diagnosis of the origin of anemia. Elevated RDW levels are frequently found in patients with anemia related to vitamin B_12_ or folate deficiency, while normal RDW can be observed in those with anemia of chronic disease or acute blood loss (7). Nowadays, a multitude of reports establish the utility of RDW as a marker of cardiovascular risk and overall mortality among patients with cardiovascular morbidities and older adults (8). However, there can be more adverse influences posed by aberrant RDW in older adults. As explained above, these patients are at risk for developing degenerative phenotypes, such as frailty. Whether high RDW levels are associated with an increased risk of frailty in older adults is rarely examined in the literature. In the current study, we investigated this issue through analyzing a large group of community-dwelling older adults.

## Methods

### Ethical Approval

The current study has been approved by the institutional review board of National Taiwan University Hospital (NO. 201802088RINC). Informed consent was deemed unnecessary by the review board due to the scrambling of participant identification at study entry and data anonymization. The study protocol adhered to the Declaration of Helsinki.

### Participant Enrollment and the Study Procedure

Community-dwelling older adults (age ≥ 65 years) were identified during their annual health examination between 2013 and 2016. For those receiving repetitive health examinations during the study period, we only recorded data from their first examination. Their baseline clinical features, including sociodemographic profiles (age, gender, habits of smoking, or alcohol consumption) and comorbidities (hypertension, DM, hyperlipidemia, gout, cardiac disorders, and laboratory-confirmed CKD [having an estimated glomerular filtration rate, eGFR < 60 mL/min/1.73 m^2^]), were recorded during examination for subsequent analysis. Following the documentation of clinical features, participants received physical examination regarding their body height, weight, waist circumference, systolic blood pressure (SBP) and diastolic blood pressure (DBP), and pulse rate. Body mass index (BMI) was calculated using weight divided by squared height. Participants received blood tests for hemogram and serum biochemistry panels including their metabolic profile, renal function, and albumin/globulin. Renal function was estimated using serum creatinine based on the Chronic Kidney Disease – Epidemiology Collaboration (CKD-EPI) formula.

For hemogram tests, 3 mL of whole blood were drawn gently from the peripheral veins, preserved in room temperature in ethylenediamine-tetraacetic acid (EDTA)-containing tubes, and sent to the central laboratory within 1 h for analysis (Automated Hematology Analyzer, Sysmex XN-10). The intra-assay and inter-assay variations were both lower than 2%. Samples with clot or hemolysis were discarded and re-sampling was carried out for accuracy. RDW was reported as the coefficient of variation of red cell volume (in percentages) relative to the MCV, as was validated and utilized by others (9–11).

### The Assessment of Frailty

The procedure for assessing frailty in this study has been outlined previously (12). Briefly, we used the Study of Osteoporotic Fractures (SOF) index to screen for frailty; that is, the presence of frailty was defined as those with ≥ 2 out of 3 items consisting of malnutrition (weight loss ≥ 5% within 3 years), poor physical performance (inability to stand up 5 times from sitting position without arm support), and fatigue (the sense of a lack of energy) (13). We further operationalized 2 of the 3 SOF items using hypoalbuminemia (<3.5 g/dL) or underweight as a surrogate for malnutrition (Quetelet index <18.5 kg/m^2^), and using the occurrence of ≥ 2 fall episodes within the preceding 6 months as a surrogate for poor physical performance (12). For those who satisfied only 1 item, they were deemed as having prefrailty. Older adults with SOF-identified frailty were reported to be at risk for having an impaired quality of life, an increased risk for disability, hospitalization and mortality (3, 4). The performance of SOF index for frailty identification is similar to other renowned frailty-screening instruments including the Cardiovascular Health Study (CHS) index and the FRAIL scale in terms of outcome correlation in different populations (14, 15).

### Statistical Analysis

We used SPSS version 19th for statistical analyses. For continuous variables, we described data in mean ± standard deviation, and compared data between groups using the Student's *t*-test (if 2 groups) and analysis of variance (ANOVA) (if > 2 groups). For categorical variables, we described data in values with percentages in parentheses, and compared data between groups using the chi-square test. These statistical tests were chosen because variables analyzed in this study were tested based on the Kolmogorov-Smirnov test.

We first divided the entire group of participants into those with a normal or a high RDW, with the threshold for determining high RDW (15.7%) based on prior studies (9, 16, 17), and compared data between those with a high and a normal RDW. We next performed univariate analyses by determining whether those with and without frailty differed regarding their clinical data, including RDW levels and the proportion of older adults with a high RDW. Finally, multiple logistic regression analyses were conducted to investigate whether having a high RDW was associated with an increased risk of frailty, incorporating significant variables (*p* < 0.05) in the univariate analysis and the RDW category. We did not select variables such as BMI and serum albumin, or variables used to calculate BMI in the multiple regression models, since these variables have been utilized to measure frailty as described above. If significant differences regarding serum creatinine or eGFR were noted in univariate analysis, we selected eGFR as the default option, since eGFR was calculated from serum creatinine and remained the standard approach of estimating renal function. We used the variance inflation factor (VIF) to estimate the degree of multicollinearity between variables in the regression models. Sensitivity analyses were done in two parts; first, we used RDW as a continuous variable and repeated the multiple regression analyses. Second, we used serum creatinine to replace eGFR in the multiple regression analyses, with results presented. In all analyses, two-tailed *p* < 0.05 were deemed statistically significant.

## Results

### Baseline Characteristics of Study Participants

During the study period, a total of 2,932 older adults (mean 73.5 ± 6.7 years; 44.6% male) were included (Table 1). One-fourth (24.3%) of our participants had a habit of regular drinking, while 4.7% has a habit of smoking. The most common comorbidity among these older adults was hypertension (48.8%), followed by cardiac diseases (20%) and hyperlipidemia (18%). Among 2,932 older adults, 113 (3.9%) had a high RDW (Table 1). Those with a high RDW had a significantly higher age (*p* = 0.002), were more likely to be diabetic (*p* = 0.013) but with a lower body weight (*p* = 0.032). Regarding hemogram, older adults with a high RDW had significantly higher RBC (*p* < 0.001) and platelet counts (*p* = 0.004) than those with a normal RDW. Serum biochemical panels showed that the high RDW group had a significantly lower serum albumin (*p* < 0.001), a higher albumin-to-globulin ratio (AGR) (*p* = 0.024), and a higher serum creatinine (*p* = 0.013) levels than those without, although eGFR did not differ between the former and the latter groups (Table 1). RDW levels correlated positively with age (*r* = 0.15, *p* < 0.001), pulse rate (*r* = 0.05, *p* = 0.01), leukocyte (*r* = 0.05, *p* = 0.01) and platelet counts (*r* = 0.06, *p* = 0.003) but negatively with AGR (*r* = −0.08, *p* < 0.001).

**Table 1:** Participants' clinical features based on RDW status.

	**Normal (*n* = 2,819)**	High^*^ (*n* = 113)	***p-value***
**Demographic profile**			
Age (years)	73.4 ± 6.7	75.3 ± 7.5	0.002
Sex (Male%)	1,251 (44)	57 (50)	0.204
**Lifestyle factors**			
Smoking (%)	129 (5)	8 (7)	0.216
Drinking (%)	689 (24)	23 (20)	0.321
**Comorbidities**			
Hypertension (%)	1,381 (49)	51 (45)	0.422
Diabetes mellitus (%)	370 (13)	24 (21)	0.013
Hyperlipidemia (%)	510 (18)	17 (15)	0.408
Chronic kidney disease (%)	292 (10)	14 (12)	0.489
Cardiac diseases (%)	565 (20)	20 (18)	0.541
Gout (%)	142 (5)	8 (7)	0.334
**Physical parameters**			
Body height (cm)	158 ± 8.2	157 ± 8.4	0.415
Body weight (kg)	59.8 ± 10.3	57.7 ± 10.6	0.032
Body mass index (kg/m^2^)	24 ± 3.4	23.3 ± 3.8	0.056
Waist circumference (cm)	83.4 ± 9.2	82.8 ± 9.9	0.489
Systolic blood pressure (mmHg)	128 ± 16.5	129 ± 19	0.446
Diastolic blood pressure (mmHg)	68.5 ± 11	68.3 ± 14.2	0.875
Pulse rate (bpm)	70.2 ± 10.8	74.2 ± 12.4	<0.001
**Hemogram**			
Red cell count (K/μL)	4.4 ± 0.5	4.9 ± 0.9	<0.001
Leukocyte (K/μL)	5.6 ± 1.5	5.7 ± 1.8	0.769
Platelet (K/μL)	210 ± 53.1	225 ± 71.8	0.004
RDW-CV (%)	13.3 ± 0.7	16.7 ± 1.3	<0.001
**Serum biochemistry**			
Albumin (g/dL)	4.29 ± 0.26	4.18 ± 0.32	<0.001
Globulin (g/dL)	2.77 ± 0.38	2.82 ± 0.43	0.181
AGR	1.54 ± 0.27	1.48 ± 0.29	0.024
Glucose (mg/dL)	101 ± 20.4	101 ± 24.4	0.699
Total cholesterol (mg/dL)	184 ± 33	170 ± 35.7	<0.001
Triglyceride (mg/dL)	119 ± 63.9	123 ± 81.8	0.555
Uric acid (mg/dL)	5.8 ± 1.4	6 ± 1.6	0.262
Creatinine (mg/dL)	0.9 ± 0.4	1 ± 0.8	0.013
eGFR (mL/min/1.73 m^2^)	84.9 ± 21.7	83.3 ± 24.3	0.454

*AGR, albumin to globulin ratio; CV, coefficient of variation; eGFR, estimated glomerular filtration rate; RDW, red cell distribution width*.

* *High RDW: RDW ≥ 15.7%*.

Importantly, we found that older adults with a high RDW level were more likely to have frailty (high vs. normal, 8 [7.1%] vs. 68 [2.4%], *p* = 0.002) and had a significantly higher number of positive SOF items than those with a normal RDW level (high vs. normal, 0.31 vs. 0.2 items, *p* = 0.013).

### Univariate Analysis: Differences in Clinical Characteristics Between Those With and Without Frailty

**Figure 1:**
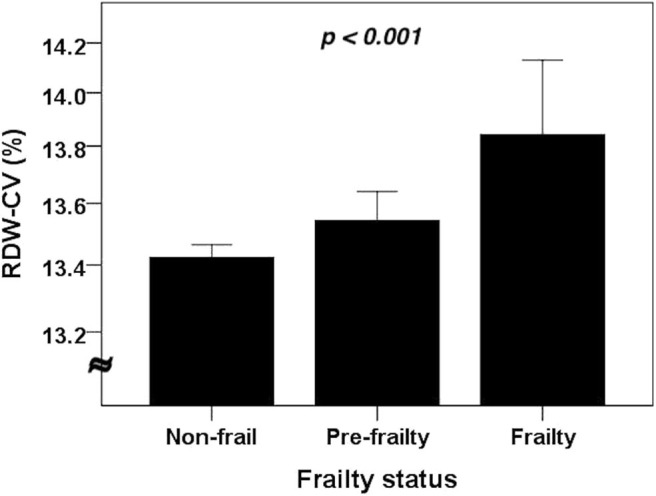
The RDW levels among older adults without and with different severities of frailty (*n* = 2,932). Compared using the analysis of variance (ANOVA). CV, coefficient of variation; RDW, red cell distribution width.

Among 2,932 older adults, 76 (2.6%) were identified as having frailty (Table 2). Frail older adults had a significantly advanced age (*p* < 0.001), more likely to have CKD (*p* = 0.004) and cardiac diseases (*p* = 0.004), a lower body weight (*p* < 0.001), waist circumference (*p* = 0.009) and BMI (*p* < 0.001) than non-frail ones. Frail older adults also had significantly lower albumin (*p* < 0.001), AGR (*p* = 0.001), and eGFR (*p* = 0.032) but higher serum creatinine levels (*p* < 0.001) (Table 2) than non-frail ones. Interestingly, frail older adults had significantly lower RBC counts (*p* < 0.001) but a higher RDW (*p* = 0.001) compared to non-frail ones. We further divided participants into those without frailty, with prefrailty (1 SOF criterion) and with frailty (> 1 SOF criterion), and compared RDW levels between each group. RDW levels increased stepwise with rising frailty severity (*p* < 0.001; Figure 1).

**Table 2:** Clinical features based on the presence of frailty or not.

	**Without (*n* = 2,856)**	**With (*n* = 76)**	***p-value***
**Demographic profile**			
Age (years)	73.3 ± 6.6	78.8 ± 8.5	<0.001
Sex (Male%)	1,280 (45)	28 (37)	0.168
**Lifestyle factors**			
Smoking (%)	134 (5)	3 (4)	0.762
Drinking (%)	704 (25)	8 (11)	0.005
**Comorbidities**			
Hypertension (%)	1,393 (49)	39 (51)	0.662
Diabetes mellitus (%)	384 (13)	10 (13)	0.942
Hyperlipidemia (%)	512 (18)	15 (20)	0.685
Chronic kidney disease (%)	291 (10)	15 (20)	0.007
Cardiac diseases (%)	560 (20)	25 (33)	0.004
Gout (%)	146 (5)	4 (5)	0.953
**Physical parameters**			
Body height (cm)	158 ± 8.2	155 ± 9.3	0.001
Body weight (kg)	59.9 ± 10.1	52.7 ± 13.8	<0.001
Body mass index (kg/m^2^)	24 ± 3.3	21.9 ± 4.9	<0.001
Waist circumference (cm)	83.5 ± 9.1	80.6 ± 12.9	0.009
Systolic blood pressure (mmHg)	128 ± 16.6	127 ± 18.2	0.44
Diastolic blood pressure (mmHg)	68.5 ± 11.1	66.1 ± 11	0.059
Pulse rate (bpm)	70.3 ± 10.8	72.6 ± 12.7	0.065
**Hemogram**			
Red cell count (K/μL)	4.5 ± 0.5	4.1 ± 0.5	<0.001
Leukocyte (K/μL)	5.6 ± 1.5	5.5 ± 1.6	0.559
Platelet (K/μL)	210 ± 53.8	214 ± 59.3	0.538
RDW-CV (%)	13.4 ± 1	13.8 ± 1.3	0.001
**Serum biochemistry**			
Albumin (g/dL)	4.3 ± 0.3	4 ± 0.3	<0.001
Globulin (g/dL)	2.8 ± 0.4	2.9 ± 0.6	0.003
AGR	1.5 ± 0.3	1.4 ± 0.3	0.001
Glucose (mg/dL)	101 ± 20.5	99.1 ± 24	0.519
Total cholesterol (mg/dL)	184 ± 33	178 ± 39.9	0.127
Triglyceride (mg/dL)	120 ± 64.7	113 ± 63	0.401
Uric acid (mg/dL)	5.8 ± 1.4	5.6 ± 1.7	0.134
Creatinine (mg/dL)	0.9 ± 0.4	1.1 ± 1.2	<0.001
eGFR (mL/min/1.73 m^2^)	85 ± 21.6	79.5 ± 28.9	0.032

*AGR, albumin to globulin ratio; CV, coefficient of variation; eGFR, estimated glomerular filtration rate; RDW, red cell distribution width*.

### Factors Independently Associated With Having Frailty

Multiple regression analyses, accounting for significant variables in univariate analysis (age, history of drinking, CKD, cardiac diseases, waist circumference, RBC, albumin to globulin ratio [AGR], and eGFR) and the RDW category, showed that higher age was independently associated with an increased risk of frailty among older adults (odds ratio [OR] 1.091, 95% confidence interval [CI] 1.054–1.129), while higher waist circumference (OR 0.962, 95% CI 0.937–0.989) and higher RBC levels (OR 0.37, 95% CI 0.229–0.598) correlated with a lower probability (original model; Table 3). We discovered that older adults with a high RDW had a significantly higher risk of frailty (OR 2.689, 95% CI 1.184–6.109) compared to those with a normal RDW (Table 3). The VIFs of the variables included in the multiple regression analyses were between 1 and 2, excluding the possibility of multicollinearity.

**Table 3:** Results from multiple logistic regression with the presence of frailty as the outcome of interest.

**Variables**	**OR**	**95% CI**	***p*-value**
Original model^*^			
Age	1.091	1.054–1.129	<0.001
CKD	1.735	1.033–2.911	0.037
Waist circumference	0.962	0.937–0.989	0.005
RBC	0.370	0.229–0.598	<0.001
High RDW	2.689	1.184–6.109	0.018
**With RDW in continuous form**			
Age	1.089	1.051–1.127	<0.001
CKD	1.706	1.016–2.865	0.043
Waist circumference	0.962	0.937–0.988	<0.001
RBC	0.375	0.233–0.604	<0.001
RDW (%)	1.223	1.023–1.461	0.027
**With serum creatinine instead of eGFR**			
Age	1.085	1.049–1.122	<0.001
Waist circumference	0.958	0.933–0.985	0.002
RBC	0.426	0.264–0.686	<0.001
High RDW	2.515	1.093–5.786	0.030

*CI, confidence interval; CKD, chronic kidney disease; eGFR, estimated glomerular filtration rate; OR, odds ratio; RBC, red blood cell; RDW, red cell distribution width*.

* *Include age, history of drinking, CKD, cardiac diseases, waist circumference, RBC count, albumin to globulin ratio, RDW category, and eGFR*.

Sensitivity analyses using RDW as a continuous variable showed that higher RDW levels were similarly associated with an increased frailty likelihood (OR 1.223 per 1% RDW higher, 95% CI 1.023–1.461), independent of other clinical features (Table 3). In addition, if we replaced eGFR with serum creatinine in multiple regression analysis, having a high RDW (OR 2.515, 95% CI 1.093–5.786) remained positively associated with the risk of frailty (Table 3).

## Discussion

In this cross-sectional study, we enrolled 2,932 community-dwelling older adults and analyzed the relationship between RDW levels and their probability of having frailty. Participants with a high RDW had a significantly higher proportion of manifesting frailty concurrently. Using multiple regression analyses, we discovered that having a high RDW level was significantly associated with a 2- to 3-fold higher risk of frailty, independent of other clinical features. This relationship persisted independent of laboratory variables related to inflammation such as AGR, as RDW has been shown to closely correlate with the severity of inflammation (18–20). Since hemogram is a convenient laboratory test during clinical practice and RDW is routinely reported in hemogram results, we believe that RDW may have potential utility as an indicator of frailty in older adults, although this finding still needs to be affirmed in other population.

Risk factors for frailty have been extensively examined in the literature. A prior review addressing the ameliorating and aggravating factors of frailty indicated that demographic features (advanced age, certain ethnicity, and having health insurance), lifestyle issues (fruit/vegetable consumption), physical factors (activities of daily living and functional status), and laboratory parameters (uric acid) were significant modifiers of frailty in older adults (21, 22). In population with a higher incidence and prevalence of frailty such as those with CKD, another systematic review also showed that demographic features, comorbidities, disability, and specific laboratory variables (albumin and testosterone) were significant risk factors for developing frailty (1). The degrees of variations of laboratory variables such as hemoglobin, total protein, creatinine, and serum phosphate have also been shown to correlate with mobility, mortality and the severity of frailty in high-risk patients (23). The observed associations between these laboratory parameters and frailty may stem from pathophysiological intermediates such as malnutrition, sarcopenia, and anemia, all of which may co-exist and contribute to the occurrence and progression of frailty (24). In this study, we enrich the existing literature by showing that RDW may be a novel laboratory indicator for frailty in relatively healthy older adults (Table 3).

The normal ranges of multiple hemogram parameters may be influenced by factors including age, gender, body composition, and ethnicity, and RDW is no exception. A higher degree of central obesity and adiposity has been shown to correlate with an increased RDW, possibly through its concurrence with insulin resistance and a low-grade inflammation (25). This is also supported by results from Furuncuoglu et al. study, which showed that BMI had a linearly positive association with RDW levels among individuals undergoing a health checkup (26). Another population-based study disclosed that RDW had an age- and gender-sensitive reference interval, with the older adults having slightly higher RDW ranges (27). In this study, we similarly showed that those with a high RDW had a significantly advanced age and were more likely to have DM (Table 1), compatible with findings from others. Moreover, our findings lend support to the prognostic importance of RDW in older adults, despite the fact that the reference range of RDW may be higher in this population.

Increased RDW is a useful marker for predicting survival and cardiovascular risk among different population, including those with heart failure (28), acute myocardial infarction (29), who were critically ill (17), with pneumonia (30), and those with malignancies (31). However, none of the existing studies identify the association between aberrant RDW levels and the risk of frailty in older adults (7). The relationship between this hemogram component and frailty in older adults may be explained by several reasons; first, the RDW-frailty association may be reflective of the biologic importance of RDW similar to that of the laboratory frailty indices, which have been shown to be important outcome predictors (32, 33). Second, a high RDW level, as a surrogate of hemogram abnormalities, may influence the possibility of having frailty through the relationship between aberrant RBC, leukocyte, or platelet indices and frailty (34–36). However, the plausibility of this reason is relatively low, since we have adjusted for RBC in our regression analyses, without any risk attenuation observed. We did not measure blood cell activities among enrollees. Finally, others have suggested that a high RDW is associated with an aggravated inflammation, a greater disease burden, and higher oxidative stress, all of which potentially underlie the mechanisms of frailty development (8, 37). However, even after we accounted for laboratory-based inflammatory parameters (AGR), the relationship between a high RDW and frailty probability stands (Table 3). In light of these findings, high RDW levels may be a surrogate for a systematically adverse health status and a bodily degenerative status. Based on the ease of obtaining RDW levels during routine patient care, we believe that RDW, besides serving as an outcome predictor, can gain new applicability as a frailty indicator in older adults.

Our study has its strengths and limitations. The phenomenon that RDW independently correlates with the risk of frailty in older adults has not been examined before, and the novelty of this topic is high. The number of our participants was satisfactory, permitting adequate statistical power to analyze determinants of our outcome of interest. However, several limitations should be born in mind. First, our participants may be representative of local community citizens only; extrapolation of our findings to other population and even population of other ethnicities should be made with caution. Second, our study is cross-sectional in nature; a causal relationship between RDW and incident frailty cannot be proven. Third, we did not measure iron profile or vitamin levels among these participants, and the influences of these variables could not be completely excluded. Nonetheless, it is more likely that aberrant RDW precedes the development of frailty. We did not identify differences in the proportion of female between those with high and normal RDW levels, contrary to results from prior studies (38). We consider that this might be related to the relatively lower proportion of older adults with a high RDW in this study. In addition, only SOF index was used to screen for frailty in our participants, which can be inadequate. Other instruments such as CHS index, FRAIL scale, or Edmonton frail scale might increase the reliability of frailty-screening results. Finally, we did not measure muscle mass, arrange body composition analysis, or assess polypharmacy in these patients (39); it can be difficult to ascertain whether these factors modify the relationship we observed.

## Conclusion

Among 2,932 community-dwelling older adults, we examined whether RDW affected the risk of having frailty. After adjusting for clinical features and laboratory parameters, a high RDW was found to be associated with a more than 2-fold higher probability of having frailty in these patients, while the magnitude of association paralleled the rising RDW levels. The mechanisms underlying this relationship may extend beyond inflammation and malnutrition only. Judging from our findings, we believe that RDW can be a promising marker for estimating the probability of having frailty among older adults.

## Data Availability Statement

The data available for this study will not be available due to institutional regulations. Requests to access the dataset can be direction to the corresponding author.

## Ethics Statement

The studies involving human participants were reviewed and approved by the institutional review board of the National Taiwan University Hospital. Written informed consent for participation was not required for this study in accordance with the national legislation and the institutional requirements.

## Author Contributions

C-ML and C-TC: study design. C-ML, C-TC, and S-IC: data analysis. C-ML, C-TC, S-IC, D-SH, and K-CH: article drafting. All authors approved the final version of the manuscript.

## Conflict of Interest

The authors declare that the research was conducted in the absence of any commercial or financial relationships that could be construed as a potential conflict of interest.

## Abbreviations

ANOVA: analysis of variance
AGR: albumin to globulin ratio
CHS: Cardiovascular Health Study
CI: confidence interval
CKD: chronic kidney disease
DM: diabetes mellitus
eGFR: estimated glomerular filtration rate
MCH: mean corpuscular hemoglobin
MDRD: Modification of Diet in Renal Disease
OR: odds ratio
RBC: red blood cell
RDW: red cell distribution width
SOF: Study of Osteoporotic Fractures.
